# Investigation of the Impact of Peri‐Implantitis Processes on Interleukin Profiles in Peri‐Implant Crevicular Fluid

**DOI:** 10.1002/cre2.70252

**Published:** 2025-12-01

**Authors:** Kristina Volchykhina, Olesya Manukhina, Ika Dewi Ana, Natalia Beshchasna, Serhii Pavlov, Oleg Mishchenko

**Affiliations:** ^1^ Department of Dentistry of Postgraduate Education Zaporizhzhia State Medical and Pharmaceutical University Zaporizhzhia Ukraine; ^2^ Dental Biomedical Sciences Department, Faculty of Dentistry Universitas Gadjah Mada Yogyakarta Indonesia; ^3^ Research Collaboration Center for Biomedical Scaffolds National Research and Innovation Agency (BRIN) – Universitas Gadjah Mada (UGM) Yogyakarta Indonesia; ^4^ Fraunhofer Institute for Ceramic Technologies and Systems IKTS Dresden Germany; ^5^ Department of Clinical Laboratory Diagnostics Zaporizhzhia State Medical and Pharmaceutical University Zaporizhzhia Ukraine

**Keywords:** biomarkers, diagnostic, immune dysregulation, interleukin (IL), peri‐implantitis (PI), targeted therapies

## Abstract

**Objectives:**

This study aimed to verify the methodology for investigation of interleukin (IL) concentrations in peri‐implant crevicular fluid (PICF) and to assess their correlation with clinical parameters, particularly bleeding on probing (BOP) and probing pocket depth (PPD).

**Material and Methods:**

PICF was collected from 60 peri‐implantitis patients and 25 healthy volunteers to analyze IL levels in both groups. Sampling was conducted using sterile dental brushes, which were placed in the peri‐implant crevice for up to 10 s. The brushes were then stored in Eppendorf tubes containing 1 mL of phosphate‐buffered saline and centrifuged at 3000 rpm for 15 min. Samples were frozen at −80°C (193,15 K) for preservation. IL concentrations were measured using enzyme‐linked immunosorbent assays (ELISA).

**Results:**

The study found significantly elevated levels of IL‐1α, IL‐6, IL‐4, and IL‐10 in PI patients compared to healthy controls. Correlation analysis revealed strong associations between BOP and IL‐1α, IL‐6, and IL‐10, while PPD showed no significant relationship with IL levels. Among these, IL‐10 had the greatest influence on BOP, highlighting its dual role in reducing excessive inflammation and sustaining chronic immune responses through immune tolerance.

**Conclusions:**

The present study demonstrated that IL‐1α, IL‐6, and IL‐10 levels in PICF are closely associated with the severity of peri‐implant inflammation. Among these cytokines, IL‐10 showed the strongest relationship with BOP, indicating its important role in the modulation of local immune activity. IL‐6 was also positively correlated with inflammatory severity, reflecting its involvement in sustaining the destructive processes within peri‐implant tissues. Overall, the obtained data suggest that IL‐6 and IL‐10 may serve as useful indicators of ongoing inflammatory activity in peri‐implantitis. However, their prognostic or diagnostic utility requires confirmation in longitudinal studies assessing temporal cytokine changes and treatment outcomes.

## Summary

- Peri‐implantitis is marked by inflammation and progressive bone loss.
- Elevated levels of interleukines have been observed in patients with peri‐implantitis.
- Elevated IL‐1α, IL‐6, IL‐4, and IL‐10 were observed in patients compared to healthy controls.
- Strong correlations were found between bleeding on probing (BOP) and IL levels.
- IL‐10 exhibited the greatest influence on BOP.

## Clinical Relevance

### Background

Peri‐implantitis (PI) is a condition characterized by inflammation and progressive bone loss around dental implants, driven by immune dysregulation. Pro‐inflammatory (IL‐1α, IL‐6) and anti‐inflammatory (IL‐4, IL‐10) interleukins play essential roles in the immune response associated with PI.

### Added Value of This Study

The obtained data emphasize the need to revise the traditional clinical‐diagnostic approach for assessing PI activity. Cytokine profiling in PICF may inform personalized diagnostic strategies and targeted therapeutic interventions for peri‐implantitis.

### Clinical Implications

Markers such as IL‐6 and IL‐10, when quantitatively evaluated in PICF, have the potential to serve as reliable biomarkers of inflammatory activity and could become the basis for personalized diagnostics and disease monitoring.

## 1 Introduction

The widespread use of dental implants has improved edentulism rehabilitation but also increased complications, with peri‐implantitis (PI) being a major concern (Fu and Wang 2020). PI results from an uncontrolled immune response to biofilm on implant surfaces, causing inflammation and bone loss (Kotsakis and Olmedo 2021), threatening implant success. Epidemiological data show a PI prevalence of 19.53% at the patient level and 12.53% at the implant level (Diaz et al. 2022), with recurrence rates of 44% and implant loss in 27% of cases (Zangani et al. 2022). Treatment options include conservative, surgical, and combined approaches, but success requires integrating clinical, structural, and pharmacological innovations (Mishchenko and Pogorielov 2019).

Early diagnosis is crucial yet challenging due to limitations in periodontal probing and radiography (Rungtanakiat et al. 2023; Lumbikananda et al. 2024). These methods detect prior destruction rather than active disease (Alassy et al. 2019). The transition from a healthy to a pathological state involves immune activation, dysregulating inflammatory mediators (cytokines, chemokines, prostaglandins), leading to tissue degradation and bone resorption (Lasserre et al. 2018).

Pro‐inflammatory (IL‐1α, IL‐6) and anti‐inflammatory (IL‐4, IL‐10) interleukins (IL) play key roles in PI. IL‐1α drives chronic inflammation and bone resorption, while IL‐6 amplifies tissue degradation (Cardoso et al. 2022). IL‐4, despite its anti‐inflammatory function, is elevated in PI sites, modulating immune responses (Giro et al. 2023). IL‐10 mitigates excessive inflammation, but its interactions require further study (Farhad et al. 2019; Li et al. 2024).

The balance between pro‐ and anti‐inflammatory mediators highlights the need for systematic research. Future studies should explore PI's molecular mechanisms to develop precise diagnostic tools. This study investigates IL‐1, IL‐6, IL‐4, and IL‐10 levels in peri‐implant crevicular fluid (PICF) to create targeted diagnostics for PI (Lumbikananda et al. 2024).

## 2 Methods

Before the study, ethical approval was obtained from the Research Ethics Committee of the Zaporizhzhia State Medical and Pharmaceutical University with the approval number: Extract from the Minutes of the Meeting of the Bioethics Commission ZSMPU №3, dated March 15, 2024. This study included patients with PI undergoing treatment at dental clinics affiliated with Zaporizhzhia State Medical and Pharmaceutical University, specifically the Exima Dental Clinic and Regional Dental Clinic. A control group of healthy individuals (patients with dental implants without peri‐implant pathology) without systemic diseases in sub‐ or decompensated stages was also formed. In total, the study involved 85 participants (aged 35–60 years), comprising 60 patients with peri‐implantitis and 25 healthy volunteers. Patients in both groups had screw‐type endosseous implants made of grade four titanium. The age distributions in both cohorts were balanced, with no statistically significant differences detected. The age of participants in the healthy volunteers group was 45.6 ± 6.9 years (35–57), and in the patient group it was 48.1 ± 8.6 years (38–60). The study included 85 participants (47 men and 38 women), with a comparable sex distribution between groups: the healthy volunteer group comprised 14 men and 11 women, while the peri‐implantitis group included 33 men and 27 women. Within the peri‐implantitis group (*n* = 60), the distribution of peri‐implantitis by number of affected implants per individual was: one implant in 40.0% (*n* = 24), two implants in 46.6% (*n* = 28), and three implants in 13.3% (*n* = 8). In cases where more than one implant was affected by peri‐implantitis, PICF was collected from a single representative implant site showing the most advanced clinical signs. All participants provided informed consent for participation and data processing. Patients in the PI group had secondary edentulism, with at least one missing tooth replaced by a fixed prosthesis supported by an implant loaded for a minimum of 12 months. Participants were enrolled after annual follow‐ups confirmed the diagnosis of peri‐implantitis.

### 2.1 Inclusion Criteria

The peri‐implantitis group included 60 participants with at least one implant affected by the condition. Diagnoses were based on clinical and radiological assessments. These included the presence of gingival inflammation (bleeding on probing [BOP] or purulent discharge during probing), radiographic evidence of bone loss ≥ 3 mm (on panoramic or targeted radiographs within 1 year of the last examination, with at least 50% of the implant surface showing osseointegration), and a probing pocket depth (PPD) ≥ 6 mm. When patients had multiple affected implants, only one implant was evaluated for consistency in the analysis.

### 2.2 Exclusion Criteria

Patients were excluded if they had taken anti‐inflammatory drugs within the preceding 2 weeks, antibiotics within the prior 3 months, or were current smokers (exclusion applied to all groups). The healthy volunteers group included 25 participants aged 35–57 years without systemic diseases or peri‐implant pathology. These individuals exhibited no signs of gingival inflammation and had probing depths ≤ 3 mm.

### 2.3 Sampling and Laboratory Analysis

PICF was collected to analyze IL levels in both groups. Sampling was conducted using sterile dental brushes, which were placed in the peri‐implant crevice for up to 10 s. The brushes were then stored in Eppendorf tubes containing 1 mL of phosphate‐buffered saline and centrifuged at 3000 rpm for 15 min. Samples were frozen at −80°C for preservation. The overall duration of biological sample collection did not exceed 6 months. After completing sample collection, all biological samples and all IL assays were analyzed by enzyme‐linked immunosorbent assays (ELISA) simultaneously in a single run. IL concentrations—specifically IL‐1α (E‐EL‐H0088, Elabscience, Wuhan, Hubei, China), IL‐4 (Cat #BMS225‐2, Invitrogen, Thermo Fischer Scientific, Waltham, Massachusetts, USA), IL‐6 (E‐EL‐H6156, Elabscience, Wuhan, Hubei, China), and IL‐10 (E‐EL‐H6154, Elabscience, Wuhan, Hubei, China) were measured using ELISA. The analysis was conducted with the ImmunoChem‐2100 system (Beckman Coulter, Brea, California, USA) on 96‐well plates coated with monoclonal antibodies targeting specific ILs. Results were quantified spectrophotometrically at 450 nm following the addition of a colorimetric reagent.

### 2.4 Statistical Analysis

Data were processed using “STATISTICA for Windows 6.0” (StatSoft Inc., License No. AXXR712D833214FAN5) and “EXCEL 7.0” (Microsoft Corp., USA). For each variable, the arithmetic mean (M) and standard error (m) were calculated. Distribution normality was assessed using the Kolmogorov–Smirnov (D), Lilliefors, and Shapiro–Wilk (W) tests. Pearson's parametric correlation method was used to calculate linear correlation coefficients (*r*), with relationships classified as weak (*r* ≤ 0.25), moderate (0.25 < *r* < 0.75), or strong (*r* ≥ 0.75). Correlations were further assessed using Spearman's rank method, with statistical significance evaluated via Student's *t*‐test and the Mann–Whitney test. Differences were considered statistically significant at *p* < 0.05.

## 3 Results

**Figure 1:**
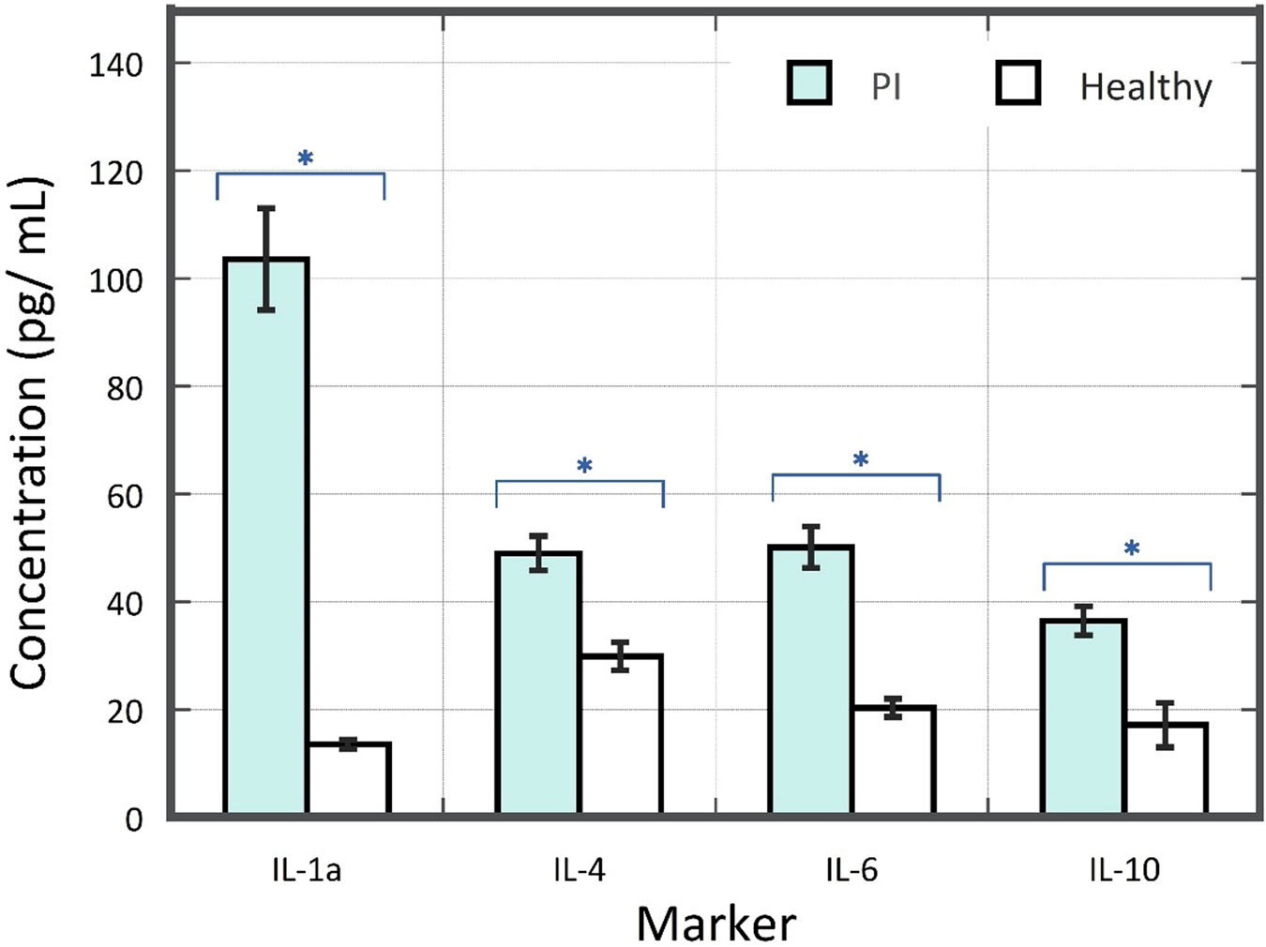
Significant increases of studied IL in patients with PI compared to healthy volunteers (*p* < 0.01).

A clinical examination of 60 patients with PI revealed that BOP was present in over 50% of sites in most cases: 66% in 34 cases and 83% in 20 cases, with an overall average of 69%. The average number of sites with a PPD ≥ 6 mm was 4.17 ± 0.16. These findings were accompanied by a statistically significant increase in the concentrations of all studied IL compared to the healthy volunteer group, as shown in Table 1. Meanwhile, results of statistical analysis on the comparability of groups and the concentration of IL between patients and healthy volunteers are shown in Figure 1.

**Table 1:** Concentration of IL‐1α, IL‐4, IL‐6, and IL‐10 in the PICF of patients with PI and healthy volunteers.

Marker	Concentration (pg/mL)		*p* value
	Patients with PI (*n* = 60)	Healthy volunteers (*n* = 25)	
Age, M ± SD	48.07 ± 8.57	45.60 ± 6.92	0.933
Sex, male, n/%	33/55	14/56	0.205
IL‐4	48.98 ± 3.21	29.87 ± 2.57	< 0.01
IL‐6	50.13 ± 3.85	20.33 ± 1.71	< 0.01
IL‐10	36.48 ± 2.65	17.11 ± 4.09	< 0.01

Correlation analysis of IL concentrations with BOP and PPD revealed that BOP showed a moderate positive correlation with IL‐1α (*r* = 0.551), IL‐10 (*r* = 0.528), and IL‐6 (*r* = 0.568). However, no correlation was observed between BOP and IL‐4 concentrations. Additionally, PPD did not show statistically significant associations with any of the IL concentrations.

To further examine the relationship between BOP and cytokine levels, a multiple regression model was constructed and validated using cross‐validation. The final model, derived after validation, is presented below:

(1) BOP=(0.258×IL−1α)+(0.290×IL−6)+(0.872×IL−10)

(2) R²=0.869,F=829.336

The cross‐validation results showed *R* ^2^ values ranging from 0.828 to 0.899 (mean *R* ^2^ = 0.869), confirming the stability of the model and the absence of overfitting. The cross‐validation slightly reduced the correlation coefficient, which is expected for biological data. However, the model retained a strong and statistically significant predictive ability, confirming that the observed relationship is stable and not driven by sample‐specific effects. This supports the biological coherence of IL‐1α, IL‐6, and IL‐10 responses during peri‐implant inflammation.

The coefficient of determination (*R* ^2^) and the *F*‐statistic demonstrated the high quality and reliability of the regression model, confirming its statistical significance and predictive power. All coefficients in the model were positive and statistically significant (*p* < 0.01). Specifically, a 10‐unit increase in IL‐1α was associated with a 2.6% increase in BOP, while a 10‐unit rise in IL‐6 resulted in a 2.9% increase in BOP. Additionally, a 10‐unit increase in IL‐10 led to an 8.7% increase in BOP.

To further investigate the relative impact of each IL on BOP, elasticity coefficients were calculated. The elasticity coefficient for IL‐1α was 0.355, for IL‐6 it was 0.222, and for IL‐10 it was 0.413. These findings indicate that IL‐10 had the most pronounced effect on BOP, highlighting its potential role as a key contributor to the observed outcomes.

**Figure 2:**
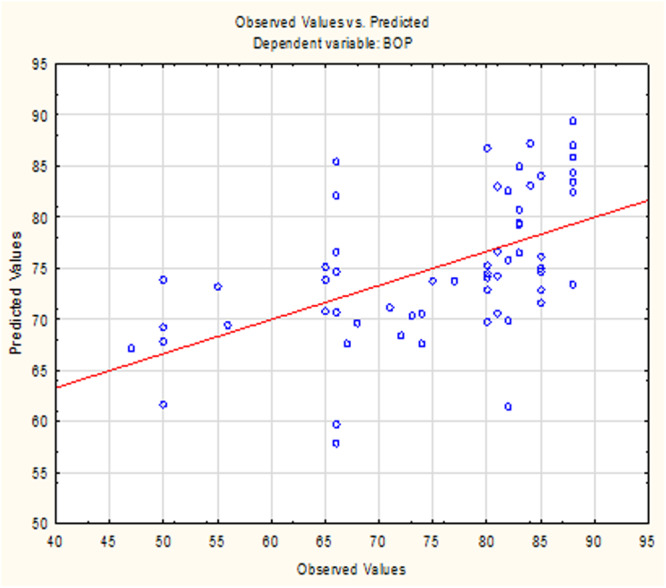
Observed and predicted values of BOP as a dependent variable. Figure visualizes the relationship between observed and predicted values of BOP based on the regression model, which evaluated the effects of interleukins IL‐1α, IL‐6, and IL‐10. The solid line represents the prediction line, where predicted values match observed values. The data points indicate individual observations, and their proximity to the solid line reflects the model's accuracy in predicting BOP. X‐axis (predicted value): Represents the predicted values of BOP based on the regression model. Y‐axis (observed value): Displays the actual observed values of BOP from the data set. Solid line: Represents the line of perfect prediction, where the predicted and observed values are identical.

The relationship between observed and predicted values of BOP is illustrated in Figure 2, which demonstrates a strong alignment between the model's predictions and the observed data points, further supporting the reliability of the regression model. The dashed lines represent the 95% confidence interval, emphasizing the precision of the model's predictions. The regression model demonstrated statistically significant positive relationships between IL levels and BOP, with IL‐10 exerting the most substantial influence based on elasticity coefficients. The close alignment of the points with the solid line and their clustering within the confidence interval supports the robustness of the model in quantifying the impact of ILs on BOP.

## 4 Discussions

The elevated concentrations IL in the PICF of patients with PI, compared to healthy individuals, reflect the activation of complex immune response cascades triggered by inflammation surrounding the implant. This inflammatory environment arises due to microbial biofilm accumulation, which stimulates host immune cells to release both pro‐inflammatory and anti‐inflammatory cytokines, thereby perpetuating the local immune response.

This study investigated the concentration of ILs (IL‐1α, IL‐6, IL‐4, and IL‐10) in PICF among patients with PI compared to healthy individuals. A significant elevation of IL‐1α, IL‐6, and IL‐10 was observed in the PI group.

Unlike previous reports that mainly described qualitative cytokine elevation in peri‐implantitis, our study introduced a quantitative regression‐based assessment linking specific IL profiles to BOP intensity. This approach allowed us to determine the relative contribution of each cytokine, revealing IL‐10 as the most influential predictor of bleeding severity. Such quantitative modeling of cytokine clinical relationships has not been previously applied in peri‐implantitis diagnostics. Moreover, by validating the model through cross‐validation, we confirmed the biological stability of the observed associations, minimizing potential data overfitting. These findings broaden the current understanding of cytokine interactions and may contribute to more precise interpretation of local immune responses in peri‐implant disease progression.

Among the key pro‐inflammatory cytokines, IL‐1α and IL‐6 play central roles. The increase in IL‐1α levels is largely attributed to the activation of immune cells, particularly macrophages, in response to microbial challenges. In this process, IL‐1α serves as a pivotal mediator in the inflammatory cascade by activating caspases, which amplify the production of inflammatory mediators. This cytokine also enhances osteoclastogenesis—the formation and activation of osteoclasts which leads to increased bone resorption around the affected implant. Furthermore, IL‐1α stimulates the synthesis of other pro‐inflammatory cytokines, such as tumor necrosis factor‐alpha (TNF‐α), creating a self‐sustaining cycle of inflammation and tissue destruction (Cardoso et al. 2022; Bertoldo et al. 2024).

The role of IL‐6 is similarly multifaceted in the context of PI. Elevated IL‐6 levels are often linked to the stimulation of acute‐phase proteins, which are key markers of systemic and local inflammation. This cytokine is involved in recruiting and activating additional immune cells at the inflammation site, thereby amplifying the inflammatory response. Notably, IL‐6 contributes to osteoclast activation and differentiation, exacerbating the process of bone resorption and leading to progressive destruction of the osseous support around the implant. These effects highlight its dual role as both a pro‐inflammatory mediator and a facilitator of tissue breakdown in peri‐implantitis (Bertoldo et al. 2024; Severino et al. 2016; Gonçalves et al. 2025). In contrast, PPD, which is traditionally used as an indicator of PI severity, showed no significant association with the concentrations of the investigated ILs. This observation suggests that PPD may primarily reflect anatomical changes rather than the current level of inflammatory activity. A similar conclusion was drawn in the study by Jansson et al. (2021).

Additionally, the persistent inflammation seen in PI reflects a failure to resolve the immune response, which typically serves to eliminate pathogens and repair tissue. Instead, the chronic presence of bacterial antigens in the peri‐implant environment prolongs the release of cytokines like IL‐1α and IL‐6, leading to a dysregulated immune response. This dysregulation not only damages connective tissue but also promotes a shift in the balance between bone resorption and formation, favoring net bone loss. Overall, the interplay between IL‐1α and IL‐6 highlights the importance of targeting these cytokines to modulate the immune response and mitigate bone resorption in PI. Further research is warranted to better understand the molecular mechanisms underlying their action and to explore therapeutic strategies aimed at controlling their effects, potentially halting the progression of PI, and at the same time providing accurate diagnostic tool concerning PI.

On the other hands, IL‐4, as a key anti‐inflammatory cytokine, plays a crucial role in modulating immune responses by activating cellular and molecular pathways that dampen inflammation and promote antibody production. This cytokine is closely linked to the activation of type 2 T‐helper cells (Th2), which secrete other anti‐inflammatory mediators such as IL‐4, IL‐5, IL‐10, and IL‐13. Collectively, these cytokines contribute to anti‐inflammatory processes by regulating macrophage activity, particularly favoring M2 macrophage polarization. This polarization reduces the production of pro‐inflammatory molecules, including TNF‐α, IL‐1α, and IL‐6, while also decreasing prostaglandin synthesis.

This study reveals that IL‐4 has dual functionality. On one hand, it helps mitigate excessive inflammation and tissue damage in the peri‐implant environment, acting as a protective factor. On the other hand, the Th2‐driven response promoted by IL‐4 may suppress other immune pathways. Specifically, this includes reduced activation of cytotoxic CD8+ T cells and natural killer cells, both of which are crucial in combating pathogenic microorganisms. This dual role could explain why no direct correlation between IL‐4 concentrations and BOP was observed in the study. While IL‐4 serves to limit inflammation, its influence on suppressing cytotoxic immune responses may allow persistent microbial challenges to exacerbate the condition.

In the context of PI, our findings regarding IL‐4 refine the current understanding of cytokine‐mediated immune regulation. Rather than functioning solely as an anti‐inflammatory mediator, IL‐4 may represent a compensatory but insufficient regulatory response that develops in parallel with the progression of tissue destruction. The increased IL‐4 levels observed in our study suggest a shift toward a Th2‐dominant immune environment that favors repair oriented rather than antimicrobial activity.

Such imbalance may suppress the Th1‐ and Th17‐associated cytokine networks essential for pathogen clearance, leading to prolonged inflammation and impaired resolution of peri‐implant lesions (Li et al. 2024).

This interpretation challenges the traditional view of IL‐4 as exclusively protective, emphasizing its context‐dependent function and its potential contribution to chronicity in peri‐implant immunopathogenesis.

It was found that elevated levels of IL‐10 in patients with PI reflect the immune system's attempt to counteract chronic inflammation surrounding the implant. It is known that IL‐10 primarily functions to suppress the overactivation of pro‐inflammatory cytokines, such as IL‐1α and IL‐6. Interestingly, the findings suggest that IL‐10 had the most significant influence on the BOP parameter. Although, in several previously published studies, IL‐10 was regarded solely as an anti‐inflammatory cytokine, reducing the severity of inflammation and contributing to the restoration of tissue homeostasis (Javed et al. 2011; Fonseca et al. 2014), its prominent effect on BOP in the context of PI may stem from multiple mechanisms. One possibility is that the heightened levels of IL‐10 represent a compensatory immune response aimed at limiting excessive inflammatory damage and protecting peri‐implant tissues from further destruction (Farhad et al. 2019; Li et al. 2024; Bertoldo et al. 2024; Martins et al. 2022).

However, elevated IL‐10 concentrations could also have paradoxical effects. In this context, IL‐10 can stimulate B cell proliferation and differentiation into plasma cells, leading to an increase in antibody production. This, in turn, may contribute to immune complex formation and secondary damage to the periodontal and peri‐implant tissues. Furthermore, excessive IL‐10 may facilitate chronic inflammation by creating an immune‐tolerant environment that allows pathogenic microorganisms to persist, and these findings are in line with recent observations (Gonçalves et al. 2025; Jansson et al. 2021; Candel‐Marti et al. 2011). By suppressing the activation of macrophages and dendritic cells, IL‐10 reduces their ability to phagocytose pathogens and limits their antimicrobial activity. In addition, IL‐10 promotes immune tolerance through the activation of regulatory T cells (Tregs), which suppress inflammatory and cytotoxic pathways. While this mechanism serves to control excessive immune reactions, it can also impair pathogen clearance, potentially prolonging the inflammatory process and allowing the disease to progress.

According to the results of this study, it can be observed that dysregulated balance between pro‐ and anti‐inflammatory cytokine production in PI was driven by intricate cellular and molecular immune interactions. The interplay between mediators such as IL‐1α, IL‐6, IL‐4, and IL‐10 alert the complexity of immune responses in PI.

These findings highlight the diagnostic relevance of IL‐6 and IL‐10 as reliable biomarkers for monitoring PI progression. In addition, to visually compare our findings with the current literature data, a comparative table (Table 2) of key peri‐implantitis biomarkers has been compiled.

**Table 2:** Comparative literature‐based biomarker table.

Marker	Conventional understanding	New findings (this study)	References
IL‐1α	Involved in bone resorption	Elevated in PI	Fonseca et al. (2014)
IL‐6	Acute inflammation marker	Elevated in PI; showed a lower correlation with BOP	Gonçalves et al. (2025) and Severino et al. (2016)
IL‐10	Anti‐inflammatory marker	Elevated in PI; possible role in chronic immune tolerance; reliably strong correlation with BOP	Jansson et al. (2021) and Gonçalves et al. (2025)
BOP	Auxiliary clinical parameter	Clearly correlated with IL‐6 and IL‐10	Gonçalves et al. (2025) and Javed et al. (2011)
PPD	Main severity indicator	Not correlated with cytokine levels	Jansson et al. (2021)

A comparative literature‐based biomarker table suggests that cytokine profiling in PICF may inform personalized diagnostic strategies and targeted therapeutic interventions for peri‐implantitis.

Thus, the obtained data emphasize the need to revise the traditional clinical‐diagnostic approach for assessing PI activity. Markers such as IL‐6 and IL‐10, when quantitatively evaluated in PICF, have the potential to serve as reliable biomarkers of inflammatory activity and could become the basis for personalized diagnostics and disease monitoring.

Based on these data, a revised approach to PI diagnostics can be proposed, incorporating comprehensive analysis of the cytokine profile in PICF as a mandatory component of clinical protocols. A promising direction includes the development of local therapeutic interventions targeting IL‐6 and IL‐10 modulation to control inflammatory activity and prevent disease progression.

## 5 Conclusions

Patients with PI exhibited a statistically significant increase in the concentrations of all studied ILs (IL‐1α, IL‐4, IL‐6, IL‐10) compared to healthy volunteers. This elevation is associated with the activation and subsequent dysregulation of immune response pathways triggered by inflammation around the implant. Such dysregulation provides a basis to understand the chronic nature of PI and its progression. Correlation analysis revealed that BOP was significantly associated with IL‐1α, IL‐10, and IL‐6. Among the studied IL, it was found that IL‐10 has the most substantial influence on BOP, thus showing its importance not only as a marker for diagnosing PI but also as a potential predictor for assessing the outcomes of treatment interventions. Oppositely, IL‐4 showed no correlation with BOP, and the PPD did not demonstrate statistically significant associations with any IL levels.

The present study demonstrated that IL‐1α, IL‐6, and IL‐10 levels in PICF are closely associated with the severity of peri‐implant inflammation. Among these cytokines, IL‐10 showed the strongest relationship with BOP, indicating its important role in the modulation of local immune activity. IL‐6 was also positively correlated with inflammatory severity, reflecting its involvement in sustaining the destructive processes within peri‐implant tissues. Overall, the obtained data suggest that IL‐6 and IL‐10 may serve as useful indicators of ongoing inflammatory activity in peri‐implantitis. However, their prognostic or diagnostic utility requires confirmation in longitudinal studies assessing temporal cytokine changes and treatment outcomes.

## Author Contributions

Conceptualization: Oleg Mishchenko. Methodology: Kristina Volchykhina and Oleg Mishchenko. Data analysis: Kristina Volchykhina, Serhii Pavlov, and Olesya Manukhina. Investigation: Kristina Volchykhina, Oleg Mishchenko, and Serhii Pavlov. Writing – original draft preparation: Oleg Mishchenko and Kristina Volchykhina. Writing – review and editing: Serhii Pavlov, Olesya Manukhina, Ika Dewi Ana, and Natalia Beshchasna. Visualization: Ika Dewi Ana and Serhii Pavlov. All authors have read and agreed to the published version of the manuscript.

## Conflicts of Interest

The authors declare no conflicts of interest.

## Data Availability

The data that support the findings of this study are available from the corresponding author upon reasonable request.
